# Evolutionary trends of reproductive phenotype in Cycadales: an analysis of morphological evolution in *Ceratozamia*

**DOI:** 10.1093/aob/mcae058

**Published:** 2024-04-27

**Authors:** Lilí Martínez-Domínguez, Fernando Nicolalde-Morejón, Francisco Vergara-Silva, David S Gernandt, Israel Huesca-Domínguez, Dennis Wm Stevenson

**Affiliations:** Posgrado en Ciencias Biológicas, Instituto de Biología, Universidad Nacional Autónoma de México, Ciudad Universitaria, 04510, Coyoacán, CDMX, Mexico; Laboratorio de Teoría Evolutiva e Historia de la Ciencia (Jardín Botánico), Instituto de Biología, Universidad Nacional Autónoma de México, Ciudad Universitaria, 04510, Coyoacán, CDMX, Mexico; Facultad de Biología, Universidad Veracruzana, Xalapa, Veracruz, 91090, Mexico; Laboratorio de Taxonomía Integrativa, Instituto de Investigaciones Biológicas, Universidad Veracruzana, 91190, Xalapa, Veracruz, Mexico; Laboratorio de Teoría Evolutiva e Historia de la Ciencia (Jardín Botánico), Instituto de Biología, Universidad Nacional Autónoma de México, Ciudad Universitaria, 04510, Coyoacán, CDMX, Mexico; Departamento de Botánica, Instituto de Biología, Universidad Nacional Autónoma de México, Ciudad Universitaria, 04510, Coyoacán, CDMX, Mexico; Instituto de Investigaciones Biológicas, Universidad Veracruzana, 91190, Xalapa, Veracruz, Mexico; The New York Botanical Garden, Bronx, NY 10458-5120, USA

**Keywords:** *Ceratozamia*, cycads, Cycadales, shape evolution, evolutionary trajectories, gymnosperm, models of character evolution, phenotype, phylogenetic signal, strobilar size, Zamiaceae

## Abstract

**Background and Aims:**

The size and shape of reproductive structures is especially relevant in evolution because these characters are directly related to the capacity for pollination and seed dispersal, a process that plays a basic role in evolutionary patterns. The evolutionary trajectories of reproductive phenotypes in gymnosperms have received special attention in terms of pollination and innovations related to the emergence of the spermatophytes. However, variability of reproductive structures, evolutionary trends and the role of environment in the evolution of cycad species have not been well documented and explored. This study considered this topic under an explicitly phylogenetic and evolutionary approach that included a broad sampling of reproductive structures in the genus *Ceratozamia*.

**Methods:**

We sampled 1400 individuals of 36 *Ceratozamia* species to explore the evolutionary pattern and identify and evaluate factors that potentially drove their evolution. We analysed characters for both pollen and ovulate strobili within a phylogenetic framework using different methods and characters (i.e. molecular and both quantitative and qualitative morphological) to infer phylogenetic relationships. Using this phylogenetic framework, evolutionary models of trait evolution for strobilar size were evaluated. In addition, quantitative morphological variation and its relation to environmental variables across species were analysed.

**Key Results:**

We found contrasting phylogenetic signals between characters of pollen and ovulate strobili. These structures exhibited high morphological disparity in several characters related to size. Results of analyses of evolutionary trajectories suggested a stabilizing selection model. With regard to phenotype–environment, the analysis produced mixed results and differences for groups in the vegetation type where the species occur; however, a positive relationship with climatic variables was found.

**Conclusions:**

The integrated approach synthesized reproductive phenotypic variation with current phylogenetic hypotheses and provided explicit statements of character evolution. The characters of volume for ovulate strobili were the most informative, and could provide a reference for further study of the evolutionary complexity in *Ceratozamia*. Finally, heterogeneous environments, which are under changing weather conditions, promote variability of reproductive structures.

## INTRODUCTION

Exploring the morphological variation of phenotype in seed plants offers an opportunity to identify and evaluate factors that drive the evolution of morphological characters (Pigliucci, 1996; Dkhar and Pareek, 2014; Weber *et al*., 2020). In particular, gymnosperm strobili appear to be relatively undiversified but there are some genera with substantial morphological differences among them and some species have fleshy structures associated with their seeds, which is a condition correlated with animal dispersal (Leslie *et al*., 2017; Nigris *et al*., 2021). Evolutionary patterns of strobilus and seed size have been studied in the largest lineage of gymnosperms, the Pinopsida (Leslie, 2011*a*; Leslie *et al*., 2014, 2017; Leal-Sáenz *et al*., 2020) but neglected in other groups. Size changes could occur also in different evolutionary pathways between pollen and ovulate strobili (Gleiser *et al*., 2019). These evolutionary trajectories are the result of different selective pressures and intricate interactions between ecological factors and developmental constraints (Leslie, 2011*a*; Gleiser *et al*., 2019).

In Cycadales, an ancient order of non-flowering seed plants, several studies, largely focused on vegetative morphologies, have shown that leaflet shape is homoplasious, and leaflet size is strongly influenced by environmental factors (Newell, 1985; Limón *et al*., 2016; Calonje *et al*., 2019). This group has separate pollen-producing and seed-producing strobili whose morphological characteristics have been of great interest because of questions related to dispersal and the change from leaf-like reproductive structures into determinate and/or indeterminate strobili in the case of ovulate plants of *Cycas* (Brenner *et al*., 2003). In addition, there are differential energetic costs in cycad reproduction because these structures are independent (Clark and Clark, 1987). Morphological characters such as strobilar and seed size, as well as the number of seeds per strobilus are indicators of the reproductive fitness of the species (López-Toledo *et al.*, 2017). However, the full range of variation in pollen and ovulate strobili has not been described and evaluated in detail (Klavins *et al*., 2013; Elgorriaga and Atkinson, 2023).

Reproductive morphology in cycads has been addressed in morphological terms as a primary resource for classification at family and generic levels (Stevenson, 1990, 1992). The incomplete and scarce preservation of reproductive structures in scientific collections and prolonged development, which lasts from 1 to 2 years, as well as a lack of knowledge of reproductive phenological patterns, has resulted in a paucity of data to close this gap (Martínez-Domínguez *et al*., 2022*a*). All these characteristics together with strobilus size are key to plant reproduction and dispersal of diaspores.

*Ceratozamia* is a genus endemic to the Mesoamerican dominion with a broad distribution in the mountainous areas of Mexico, Belize, Guatemala and Honduras (Martínez-Domínguez *et al*., 2022*b*). Currently, the diversity of this dioecious genus comprises ~36–40 species distributed in humid environments (Calonje *et al*., 2013–2024; Martínez-Domínguez *et al*., 2022*b*). Despite a rich history of research in phylogenetics (e.g. González and Vovides, 2002, 2012; Medina-Villarreal *et al*., 2019; Habib *et al*., 2023; Gutiérrez-Ortega *et al*., 2024), the morphological variation in *Ceratozamia* has long confounded systematists, with each new phylogeny seemingly producing new hypotheses of relationships. Incorporating fossil cycad taxa into phylogenetic analyses could contribute to a more robust understanding of their evolution, such as has been done for other gymnosperm groups (Leslie *et al*., 2018). The fossil record of cycads is primarily represented by vegetative structures that are often incomplete and difficult to assign to a genus (Kvaček, 2002, 2004, 2014; Hermsen *et al*., 2006; Martínez *et al*., 2012). Recent microscopy techniques have helped to clarify some fossil identities between the genera *Zamia* and *Ceratozamia*, whose macromorphological vegetative differences are inconspicuous. All fossil species of *Ceratozamia* are described based exclusively on preserved leaves. Of the four potential *Ceratozamia* species, *C. floersheimensis* (Engelhardt) Kvaček and *C. hofmannii* Ettingsh., from the lower Oligocene and the lower Miocene of Europe, respectively, can be unambiguously assigned to this genus. Unfortunately, no fossil reproductive structures of *Ceratozamia* are known to date.

In terms of shape, foliar morphological characters exhibit a high degree of homoplasy and are correlated with climatic conditions, particularly those associated with water stress (Stevenson *et al*., 1986; Medina-Villarreal *et al*., 2019). The convergence that characterizes vegetative morphology in this group has hindered phylogenetic inferences and evolution of phenotypes. The morphological stasis present in phenotypes in cycads (Renner, 2011) has discouraged the study of reproductive structures, which have been considered uninformative. Intra- and interspecific variation of strobilus size and the subsequent causes of phenotypic variation among species remain poorly understood. Surprisingly, morphological characters of ovulate strobili were correlated with annual temperature range (Martínez-Domínguez *et al*., 2022*c*). Also, evolutionary stasis is even found in the cycads genome (Wu and Chaw, 2015). The goals of this study were to analyse the evolution of the reproductive phenotype of *Ceratozamia* and test for climate influence under all environments and elevations where *Ceratozamia* species occur. We quantified variation of reproductive structures in plants that produce pollen and ovules on separate individuals, and evaluated the degree of reproductive morphological differentiation among species. We then compared the patterns of change across time and phylogenetic signal using Blomberg’s *K*, Pagel’s *λ*, and Moran’s and Abouheif’s indices. To carry out these analyses, we inferred a phylogeny with all described species except those that were described after concluding our sampling (*C. rosea* Pérez-Farr., Gut.Ortega & Vovides, *C. reesii* Vovides, Pérez-Farr. & Gut.Ortega, and *C. schiblii* Pérez-Farr. & Gut.Ortega). We also explored the distribution of characters and their contribution to the phylogenetic reconstruction.

## MATERIALS AND METHODS

### Taxon sampling

**Fig. 1.**
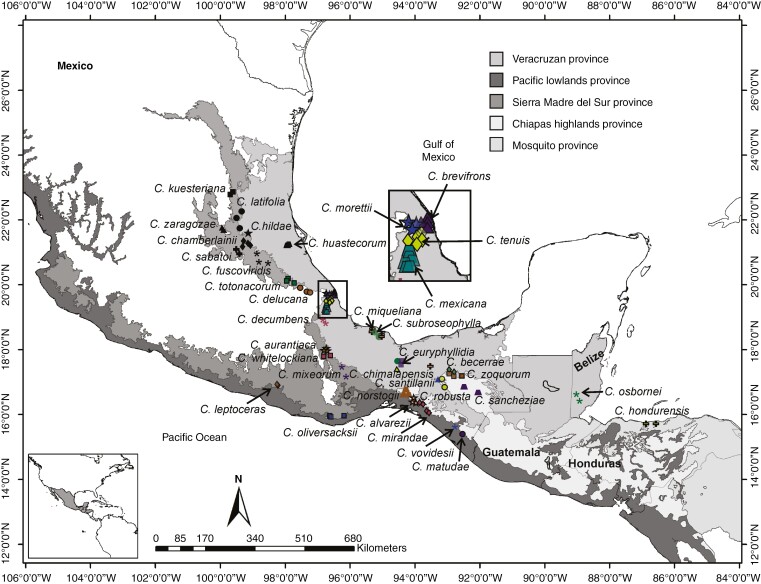
Populations of *Ceratozamia* species included in this study. The Neotropical provinces represented are according to Morrone *et al*., 2022.

This work follows the most recent circumscription of *Ceratozamia* in the monograph by Martínez-Domínguez *et al.* (2022*b*). Of the 36 species within the genus, we analysed 1500 individuals for vegetative characters from a total of 96 populations (10–20 per population) and 1400 individuals for reproductive characters from 79 populations (5–12 per population) located throughout the geographic range of the genus (Supplementary Data Table S1). Leaf samples were pressed and dried for further measurements, with all vouchers deposited in the herbaria of CIB, MEXU and TEFH. Some morphological characters were measured and coded directly in the field for each species, such as the number of leaves and the characters associated with ovulate and pollen strobili. In total, we evaluated 788 pollen strobili and 612 ovulate strobili from 76 and 70 populations, respectively. The reproductive structures were measured at the same ontogenetic stage. Thus, the pollen strobili had visible microsporangia and showed a slight separation of the microsporophylls. The megasporophylls of ovulate strobili had fully developed ovules. We developed a database with all geographic coordinates and elevations for each sampled population (Fig. 1).

### Character and character state coding

A total of 89 vegetative and reproductive morphological characters were coded as binary, multistate or a range (Supplementary Data Tables S2 and S3). We evaluated 44 morphological vegetative characters with 17 and 27 of these either quantitative or qualitative, respectively (Supplementary Data Table S2). For each reproductive structure, 22 quantitative and 23 qualitative characters were evaluated (Supplementary Data Tables S2 and S3). A qualitative character was coded as polymorphic when more than one character state occurred in a species (e.g. symmetry of leaflet lamina in *Ceratozamia delucana*). The quantitative characters for the phylogenetic analysis were expressed as intervals (mean − 1 standard error of the mean to the mean + 1 standard error) to address the diminished significance problem (i.e. the attribution of different character states to terminals when they do not differ significantly from each other; Goloboff *et al*., 2006).

### Molecular markers, sequence editing and alignment

Total genomic DNA was extracted from 100 mg of silica-dried leaflets for all *Ceratozamia* species following the protocol of the DNeasy Plant Mini Kit (Qiagen, Germantown, MD, USA). Also, we included three outgroup species: *Dioon sonorense* (De Luca, Sabato & Vázq.Torres) Chemnick, T.J.Greg. & Salas-Mor, *Bowenia serrulata* (W.Bull) Chamb. and *Zamia furfuracea* L.f. We selected nine molecular markers from nuclear and chloroplast genome regions used in the phylogenetic studies of *Ceratozamia* and other Cycadales genera (Salas-Leiva *et al*., 2013; Condamine *et al*., 2015; Medina-Villarreal *et al*., 2019; Martínez-Domínguez *et al*., 2020; Xiao *et al*., 2020). Five cycad-specific single-copy nuclear genes (SCNGs)., i.e. GroES, GTP, HTS, CyAG and PEX4; the nuclear ribosomal ITS region provided most of the phylogenetic signal in *Ceratozamia*; and three plastid DNA regions, *matK* (the maturase K coding region), *psbK/I* and *trnS-G* (intergenic spacers that have a high number of diagnostic sites)*.* PCR amplifications were performed as reported in previous published works (Shaw *et al*., 2005; Nicolalde-Morejón *et al*., 2011; Salas-Leiva *et al*., 2014). PCR products were purified using a QIAquick PCR Purification Kit (Qiagen). The PCR amplifications were evaluated by electrophoresis using 1 % agarose gel stained with ethidium bromide. Purified products were sent to Macrogen Inc. (Seoul, South Korea) for automated Sanger sequencing.

Nucleotide sequences were manually edited with Sequencher v.4.8 (Gene Codes Corp., Ann Arbor, MI, USA). Sequences for each locus were aligned using the ‘multiple alignment’ option of Clustal X in BioEdit v.7.0.9 (Thompson *et al*., 1997; Hall, 1999). The aligned lengths ranges from 615 bp (GTP) to 1254 bp (CyAG). Sequences were deposited in GenBank (Supplementary Data Table S4). Sequences of each marker were concatenated with SequenceMatrix v.1.7.8 (Vaidya *et al*., 2011).

### Phylogenetic analysis

We analysed both separate and combined matrices. Four datasets were created: (1) all morphological data, (2) all molecular data, (3) a combined matrix with morphological and molecular data of only extant species, and (4) a combined matrix with morphological and molecular data for extant and extinct species. The total length of the concatenated molecular data was 7890 nucleotide sites with no molecular data for the fossils.

All data sets were analysed using parsimony. The qualitative and molecular characters were treated as equally likely, and quantitative characters, both continuous and meristic, as additive. We carried out a heuristic search using 1000 random sequence addition replicates and TBR (Tree Bisection and Reconnection) branch swapping. Internal branches were considered unsupported and collapsed during lookups if ambiguously supported (when optimization is missing, TNT rule 1; Coddington and Scharff, 1994). For character-weighting, we used implied weighting across different constant values of concavity *K* (Goloboff, 1993, 1995; Goloboff *et al*., 2008). This ‘sensitivity analysis’ (Goloboff *et al*., 2008) was performed with *K* values of 0, 3, 10, 20, 25, 30 and 35. These values are compared with the previous results of combined data represented over the trees in the form of ‘Navajo rugs’ boxes (Wheeler, 1995). The consistency index (CI) and retention index (RI) were calculated. Two supporting indices were calculated to assess the stability of the clades, bootstrap and jackknife (36 % cutoff) with 1000 replicates. All analyses were carried out using TNT v.1.5 (Goloboff *et al*., 2008).

Simultaneous analyses using maximum likelihood (ML) and Bayesian inference (BI) with the concatenated matrix of molecular data were implemented. ML was carried out in W-IQ-TREE (Trifinopoulos *et al.*, 2016) using the ultrafast bootstrap approximation method with 1000 replicates (Hoang *et al*., 2018). Selection of the best fit model of nucleotide substitution for each dataset was carried out in ModelFinder (Kalyaanamoorthy *et al*., 2017) (Supplementary Data Table S5). Divergence times were calculated through BI using BEAST v.2.6.7 software (Drummond and Rambaut, 2007). A lognormal relaxed-clock model and a birth-death process were used as branching process priors in BEAUti (Drummond *et al.*, 2012). The calibrations were based on fossil calibrations from previous studies in Cycadales (Supplementary Data Table S6): uniform priors between 0 and 10 with a starting value of 0.1 for the mean growth rate, uniform priors between 0 and 10 with a starting value of 0.5 for the relative death rate, and an exponential prior with a mean of 0.33 on the standard deviation and a uniform prior between 0 and 1 on the mean of the model (Condamine *et al*., 2015; Calonje *et al*., 2019). The age intervals for the tree nodes were set following the 95 % age estimates reported by Condamine *et al.* (2015), Calonje *et al.* (2019) and Medina-Villarreal *et al.* (2019). Two independent runs of 50 million Markov chain Monte Carlo (MCMC) generations were performed, sampling every 1000 iterations. Log and tree files were combined using Logcombiner v.2.6.7 (included in BEAST). The log output was evaluated using Tracer v.1.7.1 (Rambaut *et al*., 2018). To elaborate a maximum clade credibility tree, we used Tree Annotator v.2.4.4 (Drummond and Rambaut, 2007), discarding 10 % of the trees as burn-in. FigTree v.1.4.4 was used to visualize the results.

The tree from BI was used to explore the evolution of reproductive morphological character state reconstruction though parsimony as implemented in Mesquite v.3.81 (Maddison and Maddison, 2023). In total, we used 11 characters considered informative and used to diagnose species within this genus, 9 of which are reproductive characters (microsporophyll shape, infertile portion shape of microsporophylls, fertile portion shape, direction of microsporophyll horns and ovulate strobilus apex shape, number of megasporophylls per row, fertile portion length of ovulate strobilus, fertile portion length of pollen strobilus and width of microsporophylls) and 2 vegetative characters (leaf colour at emergence and leaflet shape).

### Analysis of strobilus size evolution

**Fig. 2.**
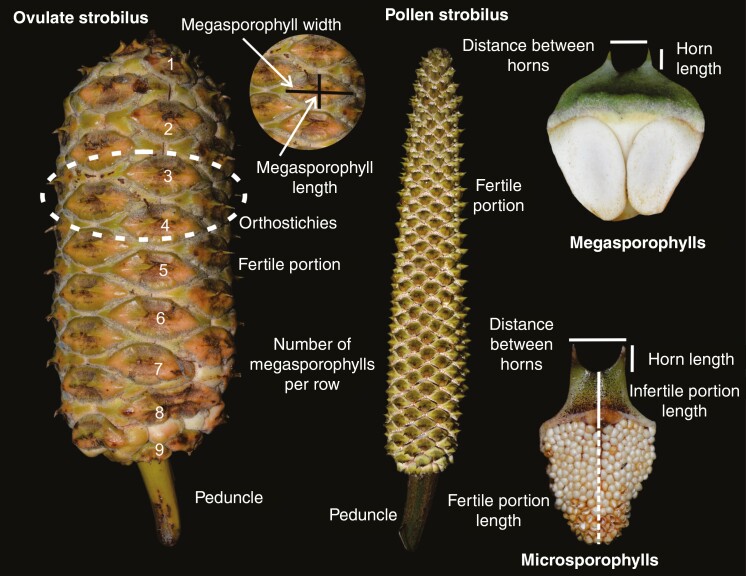
Morphology of ovulate and pollen strobili.

To explore the variation of reproductive quantitative characters between species, we evaluated all characters that included both pollen and ovulate strobili (20 reproductive characters, of which 11 correspond to the ovulate strobili and 9 to the pollen strobili). These were: (1) fertile portion length of ovulate strobilus, (2) fertile portion diameter of ovulate strobilus, (3) length of ovulate strobilus peduncle, (4) diameter of ovulate strobilus peduncle, (5) length of megasporophylls, (6) width of megasporophylls, (7) megasporophyll horn length, (8) distance between horns of megasporophylls, (9) number of megasporophylls, (10) number of orthostichies, (11) number of megasporophylls per row, (12) fertile portion length of pollen strobilus, (13) fertile portion diameter of pollen strobilus, (14) length of pollen strobilus peduncle, (15) diameter of pollen strobilus peduncle, (16) length of microsporophylls, (17) width of microsporophylls, (18) microsporophyll horn length, (19) distance between horns of microsporophylls, and (20) length of infertile portion of microsporophylls (Fig. 2). We used the ANOSIM (analysis of similarities) test to explore differences among *Ceratozamia* species, which evaluates the degree of separation among groups, generating values between −1 (differences within groups are greater than differences between groups) and 1 (higher similarity among groups than within groups). This procedure measures the overlap among the clusters. The tests were performed with 999 permutations (Clarke and Ainsworth, 1993) in the R package vegan v.2.3-5 (Oksanen *et al*., 2018).

We used four indices to test phylogenetic signal from all quantitative reproductive characters (Fig. 2). We estimated Blomberg’s *K* and Pagel’s *λ* using the function phylosig from R phytools (Revell, 2012), which are based on an explicit Brownian motion (BM) model of character evolution (Pagel, 1999; Blomberg *et al*., 2003; Münkemüller *et al*., 2012). Also, we estimated two indices of autocorrelation that do not rely directly on an evolutionary model, Moran’s *I* and Abouheif’s *C*_mean_, using the function mora.abouheif from the R package adephylo (Jombart *et al*., 2010; Münkemüller *et al*., 2012). The first describes the relation of phylogeny to cross-taxonomic character variation and considers branch length (Gittleman and Kot, 1990). The second is based on topology (not branch length) and measures autocorrelation among terminals by a phylogenetic approximation matrix (Abouheif, 1999). We made a comparison between ovulate and pollen strobili sizes for all *Ceratozamia* species. Data were scaled using min-max normalization by applying the formula *Ζ*_*i*_ = [*Χ*_*i*_ − min(*Χ*)]/[max(*Χ*) − min(*Χ*)], which scales data to a range from 1 as highest to 0 as lowest.

For each character, we tested a suite of four different evolutionary models that have been widely used in several groups, in particular, as related to phylogenetic conservatism and morphological stasis (Leslie *et al*., 2017; Glade-Vargas *et al*., 2018; Gleiser *et al*., 2019): (1) Brownian motion (BM), a stochastic model of evolution in which characters move away from the ancestral value tracking an optimum that drifts neutrally (Felsenstein, 1988); (2) Ornstein–Uhlenbeck process (OU), a stabilizing selection model of evolution with one optimum where a character evolves towards an optimal value of the ancestral character value, i.e. integrates random walk with a deterministic tendency (Harmon *et al*., 2010; Ingram *et al*., 2012); (3) early burst model (EB), a model based on a random direction of evolution of characters but with a rate of diffusion that decreases over time (Ingram *et al*., 2012); and (4) white noise (WN; non-phylogenetic) model, characterized by loss of tracks that indicate shared ancestry or random draw (Münkemüller *et al*., 2015; Gleiser *et al*., 2019). To increase the power of the test, we estimated standard errors according to our data for each species. The fitting of these models was evaluated using the Akaike information criterion (AIC), and low AIC values together with AIC weight where values nearest 1 indicate the best-fitting model (Butler and King, 2004; Diniz-Filho *et al*., 2012). The models with *Δ*AIC ≤ 2 showed more substantial support or evidence for the model with best fit (Burnham and Anderson, 2002). These analyses were performed using the R package GEIGER (Harmon *et al*., 2008).

### Relationship of reproductive quantitative characters with climate

Separately, we ran two principal component analyses (PCAs) for all populations of species and quantitative reproductive character data (Fig. 2): (1) pollen strobili, and (2) ovulate strobili. PCA allowed the visualization of reproductive morphological variation among species. A categorical variable to describe the type of vegetation for each population was created. Additionally, comparisons of the values of all reproductive characters within each vegetation type were represented using boxplots.

To evaluate the relationship of the reproductive morphological characters with climate across the range of *Ceratozamia*, we used the average values for each population, considering 19 bioclimatic variables from the WorldClim project (Hijmans *et al*., 2005; Supplementary Data Table S7) at a spatial resolution of 30 arc-sec and elevation. A redundancy analysis (RDA) was implemented with the R package vegan (Oksanen *et al*., 2018) to determine the combination of environmental variables that explain the morphological variation in both pollen and ovulate strobili. All climatic variables were standardized to the same scale. We conducted the analyses with all environment variables using R v.4.1.2 (R Core Team, 2022).

## RESULTS

### Phylogenetic analysis

The molecular matrix of only extant taxa included 7890 characters and with characters equally weighted under parsimony resulted in 27 most parsimonious trees (*L* = 2412 steps, CI = 0.831, RI = 0.781) (Supplementary Data Fig. S1A). The strict consensus was poorly resolved but the majority rule consensus tree recovered an almost completely resolved topology with three species as a polytomy (Supplementary Data Fig. S1A). The morphological matrix included 95 characters and recovered a single most parsimonious tree (*L* = 1428.617, CI = 0.417, RI = 0.570, *K* = 25) (Supplementary Data Fig. S1B). This topology has several differences in the topology of species within each of the two clades, ‘Mexicana’ and ‘Miqueliana’, when compared with the topology based on the molecular matrix (Supplementary Data Fig. S1).

**Fig. 3.**
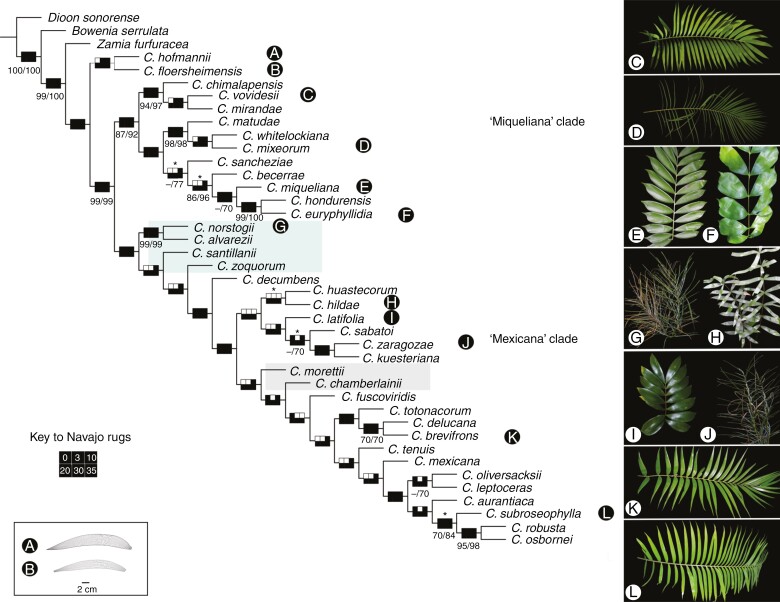
Phylogeny of *Ceratozamia* including fossil taxa based on the combined morphology (qualitative and quantitative characters) and DNA data sets. Single most-parsimonious tree recovered from a parsimony analysis under *K* = 25 (CI = 0.647, RI = 0.631, tree length = 4018.739). The groups not recovered by ML and BI omitting fossils are indicated with the coloured boxes. The support values from different resampling analysis are indicated on each branch for values >70 % (bootstrap, jackknife). Each node has a Navajo rugs (see key) summarizing the results of the sensitivity analysis applied with values *K* = 0, 3, 10, 20, 30 and 35. For details, see Supplementary Data Figs S2 and S3.

The quantitative and qualitative morphology and molecular data when concatenated included 7985 characters. The parsimony analysis had low support values, but each analysis from the implied weighting recovered only one most-parsimonious tree and the species in each of the two clades recovered were highly consistent across concavity values (*K*) with few changes in relationship among some taxa representing those clades (Fig. 3). The analysis with only extant taxa found a single most-parsimonious tree with a length of 3942.663 steps, CI = 0.659, and RI = 0.647 (*K* = 25; Supplementary Data Fig. S1B). Also, the analysis with the same data concatenated for both extant and extinct taxa found a single tree with a length of 3955.351 steps, CI = 0.657 and RI = 0.647 (*K* = 10). All reconstructions with different *K* values for this matrix showed the same clades with few changes among sister groups (Fig. 3). The topologies recovered from both analyses, with and without fossil species, were highly similar with only a few reordered relationships among sister species. *Ceratozamia*, including the two extinct taxa, was recovered as monophyletic with the two extinct species as a sister clade to the extant ones.

**Fig. 4.**
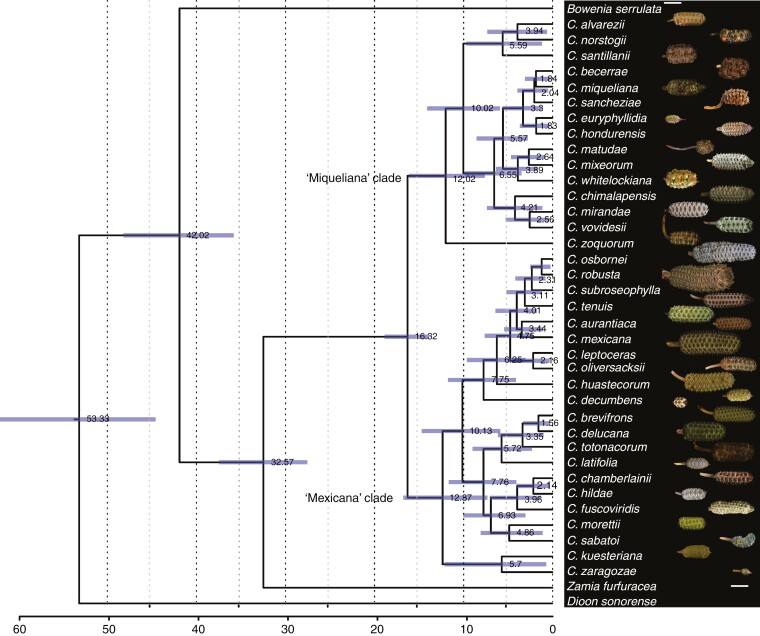
Maximum clade tree chronogram credibility obtained using single-copy nuclear genes, ITS region and plastid loci. Numbers on the branches are mean estimated ages of each clade (Ma). The purple bars indicate the 95 % highest posterior density (HPD) indices. Scale bar = 15 cm. For details, see Supplementary Data Figs S4 and S5.

All phylogenetic analyses (parsimony, ML, BI) recovered the same broad topology consisting of two major clades: (1) a ‘Miqueliana’ clade and (2) a ‘Mexicana’ clade (Figs 3 and 4; Supplementary Data Fig. S1). Most subclades were supported by >80 % bootstrap values for ML (Supplementary Data Fig. S1). Some relationships within each clade showed some discrepancies. For example, *Ceratozamia norstogii*, *C. alvarezii* and *C. santillanii* were monophyletic in the parsimony trees, but in different clades in the ML tree (Supplementary Data Fig. S1). *Ceratozamia zoquorum* was recovered in different positions and clades in separate analyses (Figs 3 and 4; Supplementary Data Fig. S1). The other species within each major clade were consistent in all the topologies.

The mean stem age of *Ceratozamia* was estimated at 32.57 Ma during the Oligocene [95 % highest posterior density (HPD) 27.62–37.57] and the crown age at 16.32 Ma (95 % HPD 13.88–18.92) during the mid-Miocene. The two major clades, ‘Miqueliana’ and ‘Mexicana’, have similar ages (Fig. 4). The split among subclades within the ‘Mexicana’ clade was estimated to be 10.13 Ma (95 % HPD 7.45–17.18 Ma) for these Sierra Madre Oriental species. Speciation within the ‘Miqueliana’ clade began ~10.02 Ma (5.91–14.09 Ma), which is consistent with the most comprehensive previous result (Habib *et al*., 2023).

### Evolution of shape: ancestral character state reconstruction

The ancestral states recovered were: (1) microsporophylls with a lobate fertile portion, (2) ovulate strobilus with an acuminate apex, (3) oblong leaflets, and (4) whitish grey trichomes on leaves. In terms of continuous characters, the reconstruction showed ovulate strobili characterized by a small size and a low number of megasporophylls per row, which in turn suggested relatively small ovulate strobili in the ancestor. Overall, the reconstruction suggested a tendency towards a higher number of megasporophylls with the total length of ovulate strobili becoming longer, but in a few species tending to have become reduced (*Ceratozamia matudae*, *C. becerrae*, *C. santillanii*, *C. zaragozae*, *C. sancheziae* and *C. zoquorum*). We found a similar size distribution for pollen strobili. The smallest and largest (extreme) sizes for microsporophylls were recovered only in the ‘Mexicana’ clade, which had the species with the fewest and widest microsporophylls (Supplementary Data Fig. S6J).

All qualitative morphological characters exhibited homoplasy (Supplementary Data Fig. S6). However, microsporophyll shape, infertile portion shape of microsporophylls, and leaflet shape revealed trends that identify subclades. The obconic shape of microsporophylls was inferred to occur almost entirely in the ‘Mexicana’ clade, whereas the elliptic shape was absent in this clade and rare in the ‘Miqueliana’ clade. An orbicular shape of the infertile portion of microsporophylls was rare in both clades and seems to have evolved separately several times; the linear shape was mainly within the ‘Miqueliana’ clade and the rounded shape was within the ‘Mexicana’ clade. With respect to vegetative characters, leaflet shape was extremely homoplasious, appearing to arise several times. The only leaflet shape character exclusive for sister species was obovate leaflets, which is a synapomorphy for *Ceratozamia hondurensis* and *C. euryphyllidia.*

### *Variation of quantitative reproductive characters among* Ceratozamia *species*

Some quantitative characters showed high correlations (>0.7) with each other (Supplementary Data Table S8). However, the characters of the sporophylls were not correlated with the characters of the strobilus total size for either pollen or ovulate strobili, with the exception of the microsporophyll width to length and diameter of the pollen strobili (Supplementary Data Table S8). Our analysis of ovulate strobili characters showed a high level of dissimilarity between *Ceratozamia* species (ANOSIM *R*-value = 0.7762) with a significance of 0.001. Most species were highly dissimilar in the size of the ovulate structures (Supplementary Data Fig. S7). Species with a high dissimilarity were *C. robusta*, *C. osbornei*, *C. matudae*, *C. decumbens* and *C. whitelockiana*. The first two species showed high similarity among themselves (Supplementary Data Fig. S7). At the same time, the rest of these species exhibited high similarity among species groups; in particular, *C. becerrae* and *C. zoquorum* were not significantly differentiated. In contrast, *C. matudae* was dissimilar to all the other species even when compared with its closely related species.

Our analysis of pollen strobili characters indicated an *R*-value closer to 1 (ANOSIM *R*-value = 0.7806) with a significance of 0.001, which suggests dissimilarity among species for characters of the pollen strobili. Several species showed high dissimilarity related to their congeners (Supplementary Data Fig. S8). In particular, *Ceratozamia osbornei* and *C. robusta* showed high segregation from several species, but high similarly which each other. Other species with high dissimilarity compared with most species within the genus were *C. leptoceras*, *C. santillanii*, *C. zaragozae* and *C. kuesteriana* (Supplementary Data Fig. S8). Overall, the pollen strobilus characters showed strong patterns of dissimilarity between sister species.

### Evolutionary patterns of reproductive structure size between ovulate and pollen strobili

**Fig. 5.**
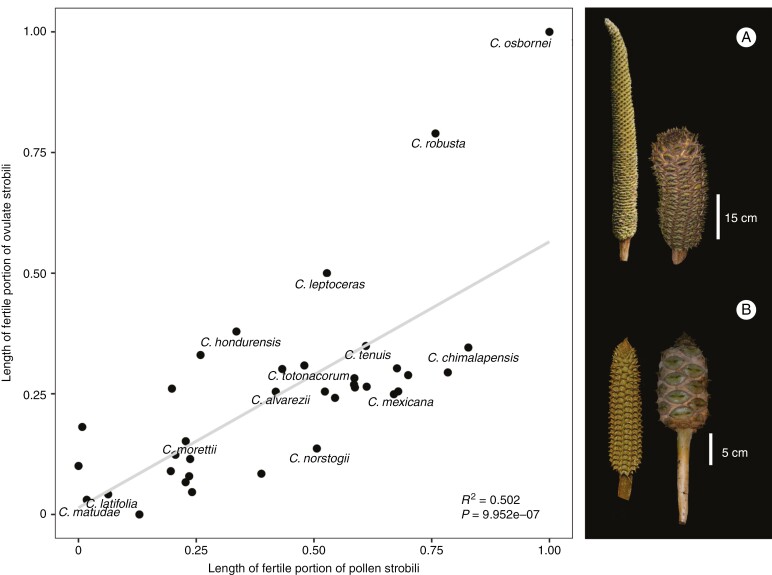
Comparisons between fertile portion length of ovulate and pollen strobili for *Ceratozamia* species. Strobili of *C. robusta* (A) and *C. latifolia* (B) on the right as examples of the disparity and similarity in sizes of ovulate and pollen strobili, respectively (Scale bars = 15 and 5 cm, respectively).

**Fig. 6.**
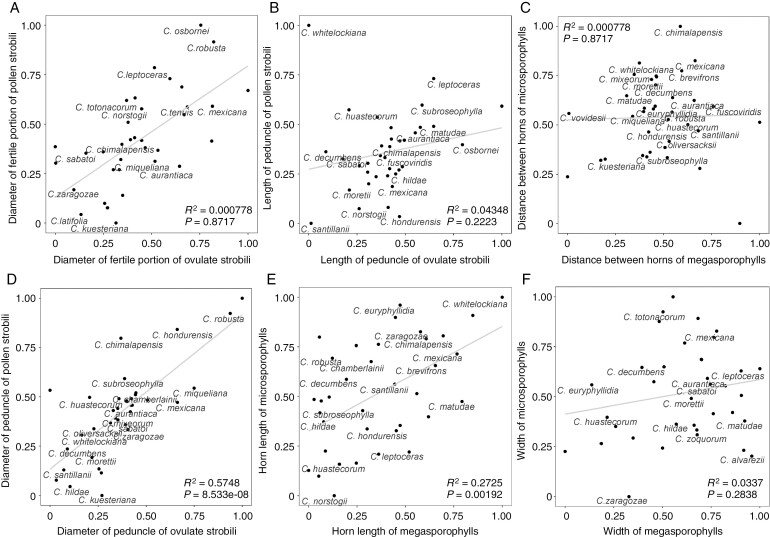
Comparison between ovulate and pollen strobili sizes for *Ceratozamia* species. (A) Diameter of fertile portion. (B) Length of peduncle. (C) Distance between horns. (D) Diameter of peduncle. (E) Horn length. (F) Width of sporophylls.

Strobilar size within *Ceratozamia* showed different evolutionary patterns when comparing pollen and ovulate strobili characters to each other (Figs 5 and 6). The length range observed in these reproductive structures overlapped in most species (Fig. 5). The relative strobilus size comparisons between the length of pollen and ovulate strobili across all species showed a positive trend whereby pollen strobilus size increases, as does ovulate strobilus size (*r* = 0.71). In both pollen and ovulate strobili, *C. matudae* and *C. latifolia* independently have the smallest strobili whereas the clade comprising *C. robusta* and *C. osbornei* have the largest strobili. Relative length variations were found in some species. This disparity was wider between ovulate and pollen strobili in those species that had long strobili. For most species, pollen strobili were longer than ovulate strobili. A few species (e.g. *C. latifolia*) had ovulate and pollen strobili of equal or proportional length (Fig. 5). The length size pattern was equal for the peduncles of both pollen and ovulate strobili in several species, including those with long strobili, such as *C. robusta* (*r* = 0.75).

Most species had similar patterns where ovulate and pollen strobili did not exhibit proportional changes in length, i.e. the strobilar size was different for pollen and ovulate strobili in the same species (Fig. 6). The diameter of pollen and ovulate strobili did show a positive trend that was proportional in a few species, such as *Ceratozamia tenuis* and *C. chimalapensis* (*r* = 0.65; Fig. 6A). In contrast, the characters directly related to the microsporophylls and megasporophylls showed the greatest size dissimilarity (Fig. 6C, E, F). This was particularly in the case of the distance between the horns of sporophylls, as shown by the clear separation from the line across all species in Fig. 6C.

Based on the inferred phylogenetic relationship, we estimated Pagel’s *λ*, Blomberg’s *K*, Abouheif’s *C*_mean_ and Moran’s *I* to determine the phylogenetic signal of pollen and ovulate strobilus size variation. In the analysis of both ovulate and pollen strobili in all species, size exhibited strong phylogenetic signals for these reproductive characters and the size of related species was more similar than expected under Brownian motion (Table 1). The width of microsporophylls had the strongest phylogenetic signal recovered under all tests (Table 1).

**Table 1.** Outcomes of the phylogenetic signal tests for all quantitative characters for pollen and ovulate strobili of *Ceratozamia* species.

Strobilus type	Quantitative character	Blomberg’s *K*	Pagel’s *λ*	Abouheif’s *C*_mean_	Moran’s *I*
		*K*/*P*	*λ*/*P*	C_mean_/SO*/*P*	Moran’s *I*/SO*/*P*
Ovulate strobilus	Fertile portion length	0.561/0.011	0.746/0.130	0.262/2.418/0.015	0.237/2.183/0.019
	Fertile portion diameter	0.387/0.161	0.192/0.288	0.124/1.138/0.139	0.108/1.256/0.117
	Peduncle length	0.416/0.118	<0.001/1	0.026/0.233/0.384	−0.009/0.215/0.375
	Peduncle diameter	0.335/0.396	<0.001/1	0.153/1.436/0.084	0.139/1.428/0.085
	Megasporophyll length	0.409/0.105	<0.001/1	0.205/1.982/0.034	0.185/1.769/0.049
	Megasporophyll width	0.291/0.677	<0.001/1	0.019/0.182/0.41	−0.003/0.270/0.354
	Megasporophyll horn length	0.392/0.1	0.074/0.645	0.205/1.894/0.036	0.185/1.849/0.048
	Distance between horns of megasporophylls	0.373/0.278	0.067/0.547	−0.042/−0.451/0.65	−0.085/−0.563/0.700
	Number of megasporophylls	0.627/0.001	0.591/0.009	0.359/3.249/0.005	0.342/3.295/0.003
	Number of orthostichies	0.606/0.001	0.565/0.010	0.340/3.079/0.006	0.326/3.162/0.001
	Number of megasporophylls per row	0.627/0.003	0.644/0.019	0.379/3.386/0.002	0.360/3.583/0.001
Pollen strobilus	Peduncle length	0.523/0.003	0.482/0.154	0.319/2.781/0.006	0.304/3.033/0.002
	Peduncle diameter	0.509/0.009	<0.001/1	0.165/1.488/0.081	0.138/1.612/0.061
	Fertile portion length	0.450/0.052	0.526/0.138	0.246/2.218/0.016	0.225/2.280/0.014
	Fertile portion diameter	0.398/0.14	<0.001/1	0.117/1.080/0.143	0.086/0.995/0.168
	Microsporophyll length	0.658/0.001	0.693/0.165	0.326/2.885/0.004	0.305/2.924/0.007
	Microsporophyll width	0.813/0.001	0.784/0.0004	0.435/3.885/0.001	0.407/3.829/0.001
	Microsporophylls horns length	0.368/0.213	0.056/0.670	0.073/0.610/0.25	0.044/0.613/0.278
	Distance between horns of microsporophylls	0.511/0.025	0.633/0.081	0.183/1.771/0.045	0.130/1.533/0.071
	Infertile portion length of microsporophylls	0.515/0.008	0.596/0.141	0.199/1.932/0.031	0.173/1.749/0.055

*SO shows deviation from random expectation.

While several ovulate strobili size characters did not show significant phylogenetic signal (Table 1), the number of orthostichies, megasporophylls and megasporophylls per row had high phylogenetic signal values. The remaining characters of ovulate strobili sizes, such as diameter of the ovulate strobilus and length of the megasporophylls, had a random distribution across the phylogeny. In relation to pollen strobili sizes, most characters showed only moderate phylogenetic signal (Table 1). The signals of length of pollen strobilus peduncle and length of microsporophylls were significantly smaller than those found for ovulate strobili, but nevertheless were recovered as relatively high values under all tests.

For strobilar size diversification, the model-based analysis using AIC weight supported OU as the best model compared with alternative evolutionary models (Table 2). Most of the size characters for ovulate strobili showed uncertainty (*Δ*AIC ≤ 2), indicating that the models EB, OU and WN were not distinguishable from each other. Only the length of ovulate strobili, number of megasporophylls per row, orthostichies and total megasporophyll characters recovered AIC values that indicated substantial support for an OU model of character evolution (Table 2). In particular, the *Δ*AIC values of diameter of the ovulate strobilus, length and diameter of ovulate strobilus peduncle, and length and width of megasporophyll characters do not provide supporting evidence to distinguish between the WN and OU models. Also, megasporophyll horn length had non-significant *Δ*AIC values between the EB and WN models (Table 2). For pollen strobilar size, the model of evolution tests showed that length of the pollen strobilus and the length of microsporophylls fitted an OU model with a weight of 0.93 and 0.71, respectively (Table 3). The length of the pollen strobilus character was indistinguishable among BM, WN and OU models, whereas width of microsporophylls was not distinguishable between BM and OU models.

**Table 2.** Parameter estimates and statistical support for evolutionary models of ovulate strobilus size characters.

Quantitative character	Parameter			Model (AIC/*Δ*AIC/AICw)			
	α	*σ* ^2^	*z*_0_	BM	EB	WN	OU
Fertile portion length	0.176	20.178	21.450	254.033/3.707/0.117	256.033/5.707/0.043	254.563/4.237/0.090	250.326/0/**0.749**
Fertile portion diameter	0.289	2.374	9.270	169.540/10.983/0.002	171.541/12.984/0.000	159.175/0.618/0.422	158.557/0/**0.574**
Peduncle length		10.559	9.192	203.536/10.316/0.003	205.536/12.316/0.001	193.220/0/**0.691**	194.867/1.647/0.303
Diameter peduncle		0.304	1.892	82.290/15.602/0.000	84.290/17.602/0.000	66.978/0.29/0.463	66.688/0/**0.536**
Megasporophyll length		0.514	2.597	90.577/7.018/0.019	92.577/9.018/0.007	83.559/0/**0.667**	85.129/1.57/0.304
Megasporophyll width		1.013	3.516	121.042/13.957/0.000	123.042/15.957/0.000	107.085/0/**0.730**	109.085/2/0.268
Megasporophyll horns length	–	–	–	−1.889/6.137/0.016	−8.026/0/**0.357**	−8.026/0/**0.357**	−7.450/0.576/0.268
Distance between horns of megasporophylls	–	–	–	47.952/6.367/0.014	41.585/0/**0.347**	41.585/0/**0.347**	41.935/0.35/0.291
Number of orthostichies	0.129	0.941	7.905	149.819/2.56/0.208	154.484/7.225/0.020	154.484/7.225/0.020	147.259/0/**0.750**
Number of megasporophylls per row	0.175	0.175	8.516	374.000/3.321/0.157	380.755/10.076/0.005	380.755/10.076/0.005	370.679/0/**0.831**
Number of megasporophylls	0.139	474.647	70.723	192.580/5.213/0.066	195.318/7.951/0.016	195.318/7.951/0.016	187.367/0/**0.899**

– Akaike weights are equal.

Bold values indicate the models with strongest support.

**Table 3.** Parameter estimates and statistical support for the evolutionary models of pollen strobilus size characters.

Quantitative character	Parameter			Model (AIC/*Δ*AIC/AICw)			
	*α*	*σ* ^2^	*z*_0_	BM	EB	WN	OU
Fertile portion length	0.254	61.539	23.719	289.512/10.293/0.005	291.512/12.293/0.002	285.337/6.119/0.044	279.218/0/**0.947**
Fertile portion diameter	0.289	1.225	4.336	141.988/8.326/0.011	143.988/10.360/0.004	135.622/1.994/0.265	133.628/0/**0.719**
Peduncle length	0.176	3.635	8.765	192.677/1.722/0.010	194.677/5.683/0.003	190.716/1.722/0.266	188.994/0/**0.720**
Peduncle diameter	0.338	0.152	1.712	65.600/11.342/0.002	67.600/13.342/0.000	55.579/1.321/0.339	54.258/0/**0.612**
Microsporophyll length	0.143	0.070	1.481	54.572/2.862/0.178	56.572/4.862/0.065	60.038/8.328/0.011	51.710/0/**0.744**
Microsporophyll width	0.105	0.016	0.981	8.306/1.823/0.256	10.306/3.823/0.094	14.849/8.366/0.009	6.483/0/**0.638**
Microsporophyll horn length		0.008	0.272	−50.586/13.013/0.001	−48.586/15.013/0.000	−63.599/0/0.730	−61.599/2/0.268
Distance between horn of microsporophylls		0.016	0.630	−36.494/3.402/0.095	−34.494/5.402/0.035	−39.896/0/**0.525**	−39.046/0.85/0.343
Infertile portion length of microsporophylls	0.194	0.010	0.486	−20.001/4.578/0.064	−18.001/6.578/0.023	−22.858/1.721/0.270	−24.579/0/**0.640**

Bold values indicate the models with strongest support.

### Strobilar size patterns associated with vegetation types

**Fig. 7.**
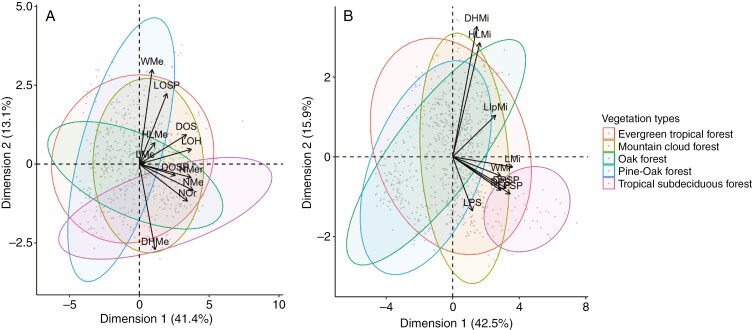
Phenotypic variation of strobili for *Ceratozamia* species by vegetation types in which the populations occurred. (A) PCA of ovulate strobili. (B) PCA of pollen strobili.

**Fig. 8.**
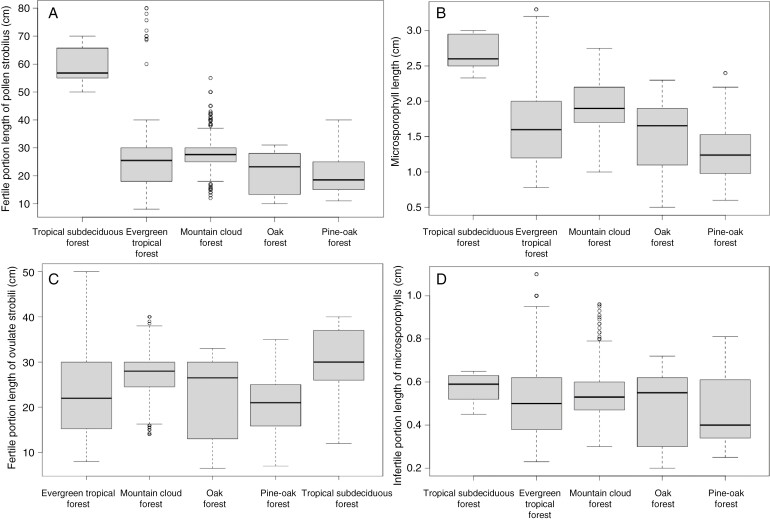
Boxplots of the morphological characters by vegetation type. (A, B, D) Characters of pollen strobilus. (C) Characters of ovulate strobilus.

*Ceratozamia* inhabits five vegetation types: evergreen tropical forest, mountain cloud forest, oak forest, pine–oak forest and tropical subdeciduous forest. Most species are restricted to one vegetation type and primarily occur in evergreen tropical forest or mountain cloud forest. There was a difference between the morphology of pollen strobili and the vegetation types, whereas in ovulate strobili there was no clear morphological separation in relation to vegetation types (Fig. 7). For the ovulate strobili, the first two components of PCA explained 64.19 % of the accumulated variance, with the higher loading values associated with characters of size in the fertile portion of the ovulate strobili (length and diameter of the ovulate strobilus, number of orthostichies and number of megasporophylls) and two megasporophyll variables (width of megasporophylls and distance between horns) (Fig. 7A; Supplementary Data Table S9). In general, ovulate strobilar size was overlapping in all the types of vegetation where most of the species occur (Fig. 7A); however, morphological variability was different within each vegetation type. Morphological variation of species in each vegetation type overlapped among different species, particularly in evergreen tropical forest, which had the widest variation. Morphological size had some differences in terms of variability range. Pine–oak forest species had the greatest variation even though few species inhabit this vegetation type. In contrast, mountain cloud forest showed the least morphological variation, although it had some extreme values that overlapped with the other vegetation types (Fig. 7A). This occurred even in the variables that most contributed to the explanation of the variation, such as in length of ovulate strobilus (Fig. 8C).

For the morphological variation of pollen strobili, 58.41 % of the accumulated variance was explained by the first two components (42.48 and 15.93 %; Supplementary Data Table S8). Distance and length of microsporophyll horns had the highest loading values (Fig. 7B; Supplementary Data Table S10). The species that occurred in tropical subdeciduous forest were clearly separated in size from species that occurred in pine–oak and oak forest as well as differing marginally from species in evergreen and mountain cloud forests (Fig. 7B). The pollen strobili of species in evergreen tropical forest showed the greatest morphological variation, whereas those in tropical deciduous forest showed the lowest variation. The mean values overlapped for the variables evaluated in most vegetation types except in tropical deciduous forest (Fig. 8). Morphological variation of species in each vegetation type showed similar sizes for variables such as length of microsporophylls and length of infertile portion of microsporophylls, and fertile portion length of pollen strobilus (Fig. 8).

### Relative contribution of climatic variables and elevation to reproductive phenotype

**Fig. 9.**
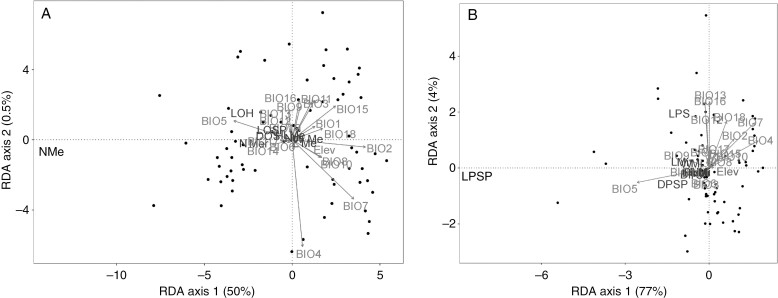
Graph of the RDAs that include both climatic variables and elevation for *Ceratozamia* species. (A) RDA of ovulate strobili. (B) RDA of pollen strobili. LPSP = length of pollen strobilus peduncle; NMe = number of megasporophylls.

Collectively, elevation and climatic variables explained 50.5 % of the total variation in the ovulate strobilar characters among the sampled species (Fig. 9A). The results of RDA for ovulate strobili showed a relationship between climatic variables and elevation and phenotypic variation of *Ceratozamia* species across their distribution range (*F* = 1.9177, *P* < 0.001, *N* = 999 permutations). This phenotype–climate interaction was not marked for a single species but rather for groups of species. The most important morphological variables were length of ovulate strobilus and number of megasporophylls (Fig. 9A); the most significant climate variables for the first axis were isothermality (BIO2) and maximum temperature of the warmest month (BIO5). For the second axis, it was temperature seasonality (BIO4) and temperature annual range (BIO7). While the largest ovulate strobili occurred in warmer areas, the variation of ovulate strobilus size was homogeneously distributed across the climatic and elevational range of all species.

The results of the full redundancy analysis for pollen strobili indicated that climatic variables and elevation had a high impact on reproductive phenotype (*F* = 9.3961, *P* < 0.001, *N* = 999 permutations). This morphological variation could correspond to the amplitude of variation observed in the vegetation types (Fig. 7). The percentage of the variance explained with two axes was 80 % (Fig. 9B). BIO4, BIO7 and BIO5 were the most significant variables for the first axis, and annual precipitation (BIO12), precipitation seasonality (BIO15), precipitation of wettest quarter (BIO16), and precipitation of warmest quarter (BIO18) the most significant for the second axis. The length of the pollen strobilus peduncle was the only character highly correlated with BIO5 (Fig. 9B). Precipitation of the wettest month (BIO13) and annual precipitation (BIO12) were variables that contributed more to the explanation of the variation of length of pollen strobilus, where this character increased only in *Ceratozamia subroseophylla*, *C. leptoceras*, *C. mixeorum* and *C. robusta*.

## DISCUSSION

### Phylogenetic relationships: fossil species

The overall topology of our phylogenetic tree mostly agrees with the last three previously published studies on *Ceratozamia* (Medina-Villarreal *et al*., 2019; Habib *et al*., 2023; Gutiérrez-Ortega *et al*., 2024; none of these included the total number of described species). The primary difference was the ‘Matudae’ clade, comprising *C. matudae* and *C. mixeorum*, which was recovered by Habib *et al.* (2023); however, a recent phylogenetic study on Cycadales by Coiro *et al.* (2023) not recovered this clade. We recovered the same two major clades as in Medina-Villarreal *et al.* (2019) with 28 taxa included, but species comprising each clade differ as well as most internal relationships. The differences could be related to the number of species in each study and number of loci included (36 species here, 30 in Habib *et al*., 2023, and 38 in Gutiérrez-Ortega *et al*., 2024). Despite the low number of loci sampled here, the relationships were highly consistent.

Incorporating fossils into a phylogenetic analysis is desirable (Donoghue *et al*., 1989; Rothwell *et al*., 2018). Nevertheless, for lineages such as *Ceratozamia*, which have a morphologically complex pattern and where extinct members are only known from characters of leaflets, it is a difficult task. Despite the ambiguity in the phylogeny recovered by the analysis of combined morphological (with fossils included) and molecular characters (Fig. 3), the position of the two fossil taxa is a clade found to be a sister group to the rest of the extant *Ceratozamia* species (Fig. 3). This result is highly consistent with a recent study that explored the origin of Cycadales incorporating species fossils into analysis in which *C. hoffmannii*, *C. floersheimensis* and *C. robusta* are being a species sister to the rest of the genus (Coiro *et al*., 2023). This disjunct distribution suggests that the genus was distributed in Europe at least until the lower Oligocene under climatic conditions of moderate to low humidity, compared with the extant species that occur in southeast Mexico (Kvaček, 2002, 2014), with its distribution becoming contracted during cool and arid periods. Considering the southern part of the Trans-Mexican Volcanic Belt as an ancestral area for *Ceratozamia* and in a humid climate (Medina-Villarreal *et al*., 2019; Habib *et al*., 2023), this could have led to the genus finally becoming extinct by the Eocene–Oligocene cooling event of temperate regions in Europe.

The vegetative macromorphological characters of *Ceratozamia* exhibit a high degree of homoplasy (Figs 3 and 4). In fact, the morphology of fossil species is most similar to the ‘Mexicana’ clade, in particular to *C. robusta*, both in shape and size of leaflets (Fig. 3), with the lanceolate shape appearing independently in both clades, with *C. vovidesii* as a clear example in the ‘Miqueliana’ clade and *C. subroseophylla* in the ‘Mexicana’ clade (Supplementary Data Fig. S6D). The fossil record and ancestral character reconstruction provided evidence of an intricate morphological evolution in *Ceratozamia* (Fig. 3) with a re-diversification beginning in the Miocene and continuing into the Pleistocene (Condamine *et al*., 2015; Habib *et al*., 2023). The climatic and geographic conditions that characterized the Trans-Mexican Volcanic Belt could be representative of geographic and ecological barriers for *Ceratozamia* dispersal (Ferrari *et al*., 2012). Multiple vicariant events at different times could result in the isolation of *Ceratozamia* populations (Martínez-Domínguez *et al*., 2022*c*) where the elevated parts of the mountainous systems of Mexico could have acted as refugia similar to that of islands during Pleistocene climatic fluctuations (Graham, 1999).

### Ovulate and pollen strobilus: diversity and evolutionary patterns

Our results showed that homoplasy was not absent in reproductive characters, but they were more phylogenetically structured (Fig. 3; Supplementary Data Fig. S6). For example, the elliptic microsporophyll shape is a novelty within the ‘Miqueliana’ clade for *Ceratozamia alvarezii*, *C. whitelockiana*, *C. mirandae* and *C. chimalapensis*, whereas the discoid and obconic shapes characterize both this clade and the ‘Mexicana’ clade. These two latter character states may have evolved independently at least three times in each clade (Supplementary Data Fig. S6C). Thus, our findings indicated that these characters across the tree could be due to both morphological stasis and parallel evolution.

On the other hand, strobilar size is a question addressed from different approaches, such as functionality in some gymnosperms, but poorly explored in cycads (Leslie, 2011*a*, *b*; Leslie *et al*., 2014; Gleiser *et al*., 2019; Martínez-Domínguez *et al*., 2022*c*). In Araucariaceae, an evolutionary transition from small to large ovulate and pollen cones with a coexistence of species that had large and small cones for a brief time was proposed using the fossil record across geological periods (Stults *et al*., 2012; Gleiser *et al*., 2019). Unfortunately, cycad strobili are scarcely represented in the fossil record and several of these records are doubtful or otherwise incomplete (Taylor, 1970; Klavins *et al*., 2013). Recently, two species were described, *Delemaya spinulosa* from the middle Triassic and *Skyttegaardia nagalingumiae* from the Late Cretaceous. These are the most complete cycad fossil pollen strobili preserved with microsporophylls helically arranged into a short pollen strobilus with a few pollen sacs per microsporophyll (Klavins *et al*., 2013; Elgorriaga and Atkinson, 2023). No pollen or ovulate strobili have been described for *Ceratozamia* even though the bicornate sporophylls are unique and easily identified for extant *Ceratozamia*. Our results showed a trend towards increased strobilus length in *Ceratozamia* (Supplementary Data Fig. S6G–K), which was clearer in ovulate strobili, both in total length and sporophyll number per column as well as the number of orthostichies (Fig. 4). Nevertheless, the exceptional preservation of the pollen strobilus of *S. nagalingumiae* indicates that this structure was mature; therefore, this small, fossil pollen strobilus and our results in turn indicate an evolution towards larger strobili in cycads (Figs 4–6; Supplementary Data Fig. S6G–K).

The dioecy of cycads, as in other gymnosperms, influences the evolution of reproductive structures and can lead to uncoupled evolution between pollen and ovulate strobili by the separate functions of diaspores (Leslie, 2011*a*, *b*; Gleiser *et al*., 2019). Cycads are pollinated by insects, and the relationships between reproductive structures depend on different signals that will undoubtedly give rise to numerous phenotypic responses where the ovulate strobilus has multifunctionality to ensure pollination and subsequently provide more protection to ovules than to pollen strobili (Terry *et al*., 2007; Salzman *et al*., 2021). Here, the size patterns between pollen and ovulate strobili for each extant species revealed coupling between the length of fertile portions for these structures in at least half of the *Ceratozamia* species (Fig. 5) and uncoupled evolution was more marked with longer strobili. The rest of the size characters evaluated, except for infertile peduncle, showed a clear uncoupled pattern (Fig. 6). Climate changes over time that have led to aridification can influence the reproductive investment in species (Liu *et al.*, 2022). This independent path followed by reproductive structures recovered in *Ceratozamia* could suggest that the large size variability in strobili is related to reproductive investment where soil nutrients and fertility, as well as phylogeny, influence the reproductive phenotype.

The results indicated more phylogenetic signal for the size of ovulate strobili than for pollen strobili (Table 1), whose evolution was best described by the OU model (Tables 2 and 3). All informative characters are related to changes in total volume of the ovulate strobilus (Tables 1–3). Under this model, ovulate strobilus size follows stabilizing selection; their phenotype may have resulted from selective pressures moving lineages towards an optimum value with little variation (Butler and King, 2004; Ingram *et al*., 2012). In contrast, pollen strobilus size shows less phylogenetic signal than ovulate strobilus size (Table 1). Only the length of the pollen strobilus and the length of microsporophylls had significant phylogenetic signal and its model was OU (Table 3). The rate of length evolution in pollen cones is substantially greater than in ovulate cones (Tables 2 and 3). We noticed that fewer variables related to volume were observed for the total number of microsporophylls during fieldwork.

### Influence of environmental heterogeneity on ovulate and pollen strobilar size

Pollen and ovulate strobilar sizes of *Ceratozamia* have been considered less variable than vegetative characters such as width or length of leaves and leaflets (Whitelock, 2004). Here, we found high variability of pollen and ovulate strobilar size at intra- and interspecific levels for most *Ceratozamia* species (Supplementary Data Figs S7 and S8). The variational properties of pollen and ovulate characters differed markedly, and characters of pollen strobili were more variable than those in ovulate strobili. Variation in pollen and ovulate strobili size by vegetation type cannot be explained by a direct correlation (Figs 7–9). However, the patterns showed a tendency to more variability in strobilus size for heterogeneous environments where the genus occurs, such as evergreen tropical forest with higher temperatures and strong seasonal precipitation (Miranda and Hernández-X, 1963). Some characters, such as the length of microsporophylls and the length of the pollen strobilus peduncle, showed atypical values and the widest range of variation. Thus, the longest ovulate strobili were in regions with the highest maximum temperature during the warmest month (Figs 7 and 9).

Overall, the results suggested an influence of climate on strobilus size, but the relationship was difficult to explain because we observed a mixed pattern without an apparent ecological trend for all species (Fig. 9). The phenotype–climate interaction does not represent a generality for all *Ceratozamia* species. An increase in number of megasporophylls or length of ovulate strobilus is correlated with warmer regions, but only for some species (Fig. 9A). The species inhabiting mountain cloud forest showed less variability (i.e. a moderate range with respect to the total variability recorded within this genus; Fig. 8). In contrast, pollen strobilus variability was not directly related to climate and elevation for most species. The range of morphological variability was similar in different habitat types with a clear tendency to larger pollen strobili and microsporophylls in species that occurred in tropical sub-deciduous forest (Figs 8 and 9). Nevertheless, the amplitude of morphological variability in mountain cloud forest is similar in both ovulate and pollen strobili, which was characterized by a constant and similar range for each species. This habitat exhibits homogeneous levels of humidity that are more persistent despite the presence of a dry and cold period during the year that is maintained by the clouds that usually form (Williams-Linera *et al*., 2013). The similar sizes in the different species could be related to the constant humidity, whereas the other habitats have regimes that change throughout the year.

The variability of environments could drive changes in reproductive phenotypes. In particular, environmental heterogeneity where seasonal fluctuations operate would provide selective pressure (Schurr *et al*., 2008; Fusco and Minelli, 2010; Pélabon *et al*., 2013). In *Pinus*, the morphology associated with dispersal is related to the extremes of seasonal fluctuations such as temperature and precipitation (Salazar-Tortosa *et al*., 2019). We found broader phenotypic reproductive variation for *Ceratozamia* in those environments with more fluctuations (Figs 8 and 9). Heterogeneous environments would have an indirect effect on reproductive phenotypes only by promoting or generating variation. On the other hand, a large strobilar size variation has been reported both within and among populations of *Ceratozamia* in species with narrow leaflets from Soconusco in Mexico (Martínez-Domínguez *et al*., 2022*c*). Another species with this variation, but only in ovulate strobili, was *C. aurantiaca*, which has ovulate strobili from 11 to 40 cm long and 4–19 sporophylls per row (Fig. 7; Martínez-Domínguez *et al*., 2022*d*). Some fossils of *Androstrobus* support the variability in pollen strobili of cycads (Harris, 1941). This high variability in strobilar size could be a response mechanism to different environmental pressures. When plasticity of phenotype is adaptative, plants are able to respond to the adverse effects of different environmental conditions (Chevin and Hoffmann, 2017).

### Conclusions

Our findings provide insights into the evolutionary trajectories shaping strobilus sizes that may have occurred by natural selection. The size characters with the most phylogenetic signal were related to strobilus volume (Table 1). We did not directly assess the volume of these structures; therefore, future studies developing or applying methods to measure the total volume of structures could be relevant to address the still open questions of evolutionary trajectories. A total decoupling between pollen and ovulate strobili would be expected (Leslie, 2011a; Gleiser *et al*., 2019); however, we found that several species were the same in the relative length of the complete strobili (Fig. 5), but quite different in their ovulate and pollen reproductive units (i.e. characters of sporophylls; Fig. 6). The exploration of shapes for these structures provides complementary evidence for characterizing the ancestral characters within this genus.

The larger and shorter strobili in *Ceratozamia* occur in habitats characterized by wider variation in climate conditions. The species with low intra- and interspecific variability with the mainly shorter strobili occur in drier habitats, whereas longer strobili are found in temperate habitats. Conversely, species with high variability at the intra- and interspecific level occupy a broader climatic range and seasonality. Thus, climate may act as a promoter in shaping variability in strobilus size, as has been reported for seed size in other gymnosperms (Leslie *et al*., 2017). We consider that the strobilus size could be the result of multiple ecological and developmental factors, but not undergoing by morphological stasis. The cycads have been considered to be an evolutionary dead-end because they are strictly coevolved and highly specialized systems, and they underwent genome duplication (Vamosi *et al*., 2014; Roodt *et al*., 2017); however, the higher levels of plasticity reported in this group might allow preadaptations to extreme conditions at different spatial and temporal scales. Finally, these data and results were obtained for *Ceratozamia*. The conclusions for cycads in general could be tested in other genera, such as *Dioon* and *Macrozamia*, for which we have modern monographs and molecular data.

## SUPPLEMENTARY DATA

Supplementary Data are available at *Annals of Botany* online and consist of the following.

Table S1: sampled populations. Table S2: qualitative morphological characters and their respective character states (vegetative and reproductive). Table S3: quantitative morphological characters and abbreviations (vegetative and reproductive). Table S4: primers used in this study and GenBank accession numbers. Table S5: molecular markers best-fit evolutionary model for trees inferred from Bayesian and Maximum Likelihood analyses. Table S6: selected mean clade ages (millions of years ago) and their 95 % HPD intervals. Table S7: bioclimatic variables and abbreviations according to WorldClim. Table S8: correlations among the quantitative morphological characters. Table S9: principal component analysis for all quantitative characters of the ovulate strobili in *Ceratozamia* species. Table S10: PCA for all quantitative characters of the pollen strobili in *Ceratozamia* species. Figure S1: (A) majority rule consensus tree of *Ceratozamia* in parsimony based on DNA data; (B) single most parsimonious tree based on all morphological data and recovered from parsimony analysis under *K* = 25. Figure S2: reconstruction of *Ceratozamia hofmannii*. Figure S3: reconstruction of *Ceratozamia floersheimensis*. Figure S4: ovulate strobili of *Ceratozamia*. Figure S5: ovulate strobili of *Ceratozamia*. Figure S6: ancestral character state reconstruction. Figure S7: heat map showing the results of analysis of similarity (ANOSIM) of quantitative characters in ovulate strobili for *Ceratozamia* species along with a dendrogram heat map of each sample. Figure S8: heat map showing the results of analysis of similarity (ANOSIM) of quantitative characters in pollen strobili for *Ceratozamia* species along with a dendrogram heat map of each sample.

mcae058_suppl_Supplementary_Tables_S1_S10

mcae058_suppl_Supplementary_Figures

mcae058_suppl_Supplementary_Tables_S8

## Data Availability

The data presented in the article and Supplementary Data should be enough for reproducing our results; raw data are available from the corresponding author upon request.
