# Supplementary material for: Evolutionary trends of reproductive phenotype in Cycadales: an analysis of morphological evolution in Ceratozamia
Source: Ann Bot. 2024 Apr 27;134(4):631–50. doi: 10.1093/aob/mcae058 (PMC11523635; doi:10.1093/aob/mcae058)
Supplement: mcae058_suppl_Supplementary_Tables_S1_S10 [file mcae058_suppl_supplementary_tables_s1_s10.docx]

Manuscript: Evolutionary trends of reproductive phenotype in Cycadales: an analysis of morphological evolution in *Ceratozamia*

Authors: Lilí Martínez Domínguez, Fernando Nicolalde Morejón, Francisco Vergara Silva, David S. Gernandt, Israel Huesca Domínguez, Dennis Wm. Stevenson.

The matrices of data such as nexus, TNT, and XML are available upon request to the authors.

**SUPPLEMENTARY DATA**

Supplementary Data Table S1. Sampled populations. The * indicates number of populations with pollen and/or ovulate strobili.

| Species | Populations |
| --- | --- |
| *C. alvarezii* | 3 (2*) Cintalapa, Chiapas, Mexico |
| *C. aurantiaca* | 3 (2*) San Pedro Teutila, Oaxaca, Mexico |
| *C. becerrae* | 1* Tacotalpa, Tabasco, Mexico; 1* Teapa, Tabasco, Mexico |
| *C. brevifrons* | 2* Alto Lucero de Gutiérrez Barrios, Veracruz, Mexico; 2* Chiconquiaco, Veracruz, Mexico |
| *C. chamberlainii* | 1* Xilitla, San Luis Potosí, Mexico; 1* Chapulhuacán, Hidalgo, Mexico; 2* Landa de Matamoros, Querétaro, Mexico |
| *C. chimalapensis* | 1* Santa María Chimalapa, Oaxaca, Mexico |
| *C. decumbens* | 1* Coetzala, Veracruz, Mexico; 1* Naranjal, Veracruz, Mexico |
| *C. delucana* | 1* Atzalan, Veracruz, Mexico; 1* Landero y Coss, Veracruz, Mexico; 1* Xochitlán de Vicente Suárez, Puebla, Mexico |
| *C. euryphyllidia* | 1 Uxpanapa, Veracruz, Mexico; 1* Santa María Chimalapa, Oaxaca, Mexico |
| *C. fuscoviridis* | 1* Molando de Escamilla, Hidalgo, Mexico; 1 Tlanchinol, Hidalgo, Mexico; 1 *Zacualtipán de Ángeles, Veracruz, Mexico |
| *C. hildae* | 1* Aquismón, San Luis Potosí, Mexico |
| *C. hondurensis* | 1* La Ceiba, Atlántida, Honduras; 1* Tela, Atlántida, Honduras |
| *C. huastecorum* | 2* Tepetzintla, Veracruz, Mexico |
| *C. kuesteriana* | 2 (1*) Tula, Tamaulipas, Mexico |
| *C. latifolia* | 1* Naranjo, San Luis Potosí, Mexico; 1* Rayón, San Luis Potosí, Mexico; 1* Tamasopo, San Luis Potosí, Mexico |
| *C. leptoceras* | 2* Tlacoachistlahuaca, Guerrero, Mexico |
| *C. matudae* | 1* Acacoyagua, Chiapas, Mexico |
| *C. mexicana* | 1* Comapa, Veracruz, Mexico; 2* Teocelo, Veracruz, Mexico; 1* Tlaltetela, Veracruz, Mexico; 2* Totutla, Veracruz, Mexico; 1 Xico, Veracruz, Mexico |
| *C. miqueliana* | 1 Huimanguillo, Tabasco, Mexico; 2 (1*) Ángel R. Cabada, Veracruz, Mexico; 2 (1*) Santiago Tuxtla, Veracruz, Mexico |
| *C. mirandae* | 1 Jiquipilas, Chiapas, Mexico; 3* Villaflores, Chiapas, Mexico |
| *C. mixeorum* | 1 Santa María Tepantlali, Oaxaca, Mexico; 1* San Juan Juquila Mixes, Oaxaca, Mexico |
| *C. morettii* | 2* Chiconquiaco, Veracruz, Mexico; 1* Landero y Coss, Veracruz, Mexico |
| *C. norstogii* | 1* Santo Domingo Zanatepec, Oaxaca, Mexico; 2* Cintalapa, Chiapas, Mexico |
| *C. oliversacksii* | 1* Candelaria Loxicha, Oaxaca, Mexico; 1 San Agustín Loxicha, Oaxaca, Mexico; 1 San Miguel del puerto, Oaxaca, Mexico |
| *C. osborneii* | 1* Cayo, Belize; 1* Toledo, Belize. From herbarium specimens and cultivated plants |
| *C. robusta* | 1* Berriozábal, Chiapas, Mexico; 1* Tuxtla Gutiérrez, Chiapas, Mexico |
| *C. sabatoi* | 1* Cadereyta de Montes, Querétaro, Mexico; 1* Pinal de Amoles, Querétaro, Mexico |
| *C. sancheziae* | 1* Altamirano, Chiapas, Mexico; 1* Tenejapa, Chiapas, Mexico; 1 |
| *C. santillanii* | 1* Berriozábal, Chiapas, Mexico |
| *C. subroseophylla* | 1* Uxpanapa, Veracruz, Mexico; 2* Santiago Tuxtla, Veracruz, Mexico |
| *C. tenuis* | 1* Jilotepec, Veracruz, Mexico; 1 Xalapa, Veracruz, Mexico; 1* Coacoatzintla, Veracruz, Mexico; 1* Chiconquiaco, Veracruz, Mexico; 2* Tepetlán, Veracruz, Mexico |
| *C. totonacorum* | 1 Cuetzalan del Progreso, Puebla, Mexico; 2* Jonotla, Puebla, Mexico |
| *C. vovidesii* | 1* La Concordia, Chiapas, Mexico |
| *C. whitelockiana* | 1* San Juan Bautista Valle Nacional, Oaxaca, Mexico; 1 Santiago Comaltepec, Oaxaca, Mexico |
| *C. zaragozae* | 1* Río Verde, San Luis potosí, Mexico |
| *C. zoquorum* | 3* Solosuchiapa, Chiapas, Mexico*; 1 Tila, Chiapas, Mexico |
|  |  |

Supplementary Data Table S2. Qualitative morphological characters and their respective character states (vegetative and reproductive).

| Character | Character state |
| --- | --- |
| Stem type | (0) hypogeous; (1) semi-hypogeous; (2) epigeous |
| Prickles on petiole | (0) absent; (1) present |
| Prickles on rachis | (0) absent; (1) present |
| Prickles type | (0) thin; (1) robust |
| Trichomes color | (0) whitish gray; (1) brown; (2) reddish-brown; (3) brownish yellow |
| Leaf color at emergence | (0) light-green; (1) dark-brown; (2) brown; (3) yellowish green; (4) reddish brown; (5) darkgreen; (6) green; (7) golden brown |
| Leaf position | (0) ascending; (1) descending |
| Insertion of leaflets on the rachis | (0) keeled; (1) plane |
| Arrangement of leaflets on the rachis | (0) opposite; (1) sub-opposite; (2) clustered |
| Arrangement of petiole | (0) straight; (1) twisted |
| Arrangement of rachis | (0) straight; (1) twisted |
| Leaflet shape | (0) obovate; (1) lanceolate; (2) oblong; (3) linear; (0) oblanceolate |
| Leaflet direction | (0) planar; (1) abaxially curved |
| Symmetry of leaflet lamina | (0) basally falcate; (1) not basally falcate |
| Leaflet consistency | (0) coriaceous; (1) papyraceous; (2) membranaceous |
| Symmetry of leaflet apex | (0) symmetric; (1) asymmetric |
| Venation appearance | (0) non-conspicuous; (1) conspicuous; (2) prominent |
| Color of leaflet base | (0) green; (1) yellow; (2) brown; (3) greenish |
| Leaflet apex shape | (0) acuminate; (1) acute; (2) rounded; (3) slight pungent |
| Leaflet base shape | (0) attenuate; (1) cuneate |
| Glaucous leaflet color (adaxial side) | (0) absent; (1) present |
| Glaucous leaflet color (abaxial side) | (0) absent; (1) present |
| Imbricate leaflets | (0) absent; (1) present |
| Involution of leaflet lamina | (0) flat; (1) canaliculate; (2) lightly involute; (3) strong canaliculate |
| Pollen strobilus position | (0) erect; (1) pendulous |
| Pollen strobilus shape | (0) angulate; (1) cylindrical |
| Pollen strobilus color | (0) greenish yellow; (1) greenish brown  (2) reddish brown; (3) yellowish brown; (4) Wine; (5) beige; (6) white |
| Distal face of microsporophylls | (0) non-recurved; (1) recurved; (2) reflexed |
| Microsporophylls shape | (0) discoid; (1) obconic; (2) elliptic; (3) rhomboid |
| Microsporophylls horns type | (0) thin; (1) robust |
| Infertile portion shape of microsporophylls | (0) orbicular; (1) rounded; (2) linear; (3) triangular |
| Fertile portion shape of microsporophylls | (0) lobate; (1) deeply lobate; (2) straight |
| Direction of horns microsporophylls | (0) straight; (1) recurved |
| Angle between horns of microsporophylls | (0) acute; (1) obtuse; (2) right |
| Ovulate strobilus position | (0) erect; (1) pendulous |
| Ovulate strobilus color (trichomes) | (0) light brown; (1) blackish to dark; (2) brown; (3) reddish-brown; (4) greyish gray; (5) wine; (6) pink; (7) whitish gray |
| Ovulate strobilus apex | (0) acuminate; (1) acute; (2) mucronate; (3) apiculate; (4) aristate |
| Ovulate strobilus shape | (0) cylindrical; (1) globose; (2) ovoid |
| Angle between horns of megasporophylls | (0) acute; (1) right; (2) obtuse |
| Megasporophylls horns type | (0) thin; (1) robust |
| Direction of horns megasporophylls | (0) non-recurved; (1) recurved |
| Distal face of megasporophylls type between horns | (0) truncate; (1) prominent |
| Sarcotesta color | (0) whitish yellow; (1) whitish red; (2) red; (4) yellow |
| Seed shape | (0) ovate; (1) globose; (2) spherical; (3) ovoid |
| Distal end of megasporophylls type | (0) concave; (1) straight; (2) bicornate |
| Distal face of megasporophylls shape | (0) triangular; (1) hexagonal |
| Margin of leaflets type | (0) entire; (1) denticulate |
| Articulation of leaflets type | (0) plane; (1) prominent; (2) decurrent |
| Distal end of leaflet margin | (0) entire; (1) sinuate |
| Distal end of microsporophylls type | (0) concave; (1) straight; (2) bicornate |
| Visible veins on adaxial side of leaflets | (0) absent; (1) present |
|  |  |
| Trichomes on distal end of megasporophylls | (0) scarcely trichomes; (1) pubescent; (2) tomentose |
| Trichomes on distal end of microsporophylls | (0) scarcely trichomes; (1) pubescent; (2) tomentose |
| Direction of distal end of microsporophylls | (0) curved; (1) rounded; (2) right |
| Infertile portion on apex of ovulate strobili | (0) absent; (1) present |
| Infertile portion on apex of pollen strobili | (0) absent; (1) present |

Supplementary Data Table S3. Quantitative morphological characters and abbreviations (vegetative and reproductive).

| Character | Abbreviation |
| --- | --- |
| Number of leaves | NL |
| Pairs of leaflets | PL |
| Length of petiole | LP |
| Length of rachis | LR |
| Length of basal leaflet | LBL |
| Width of basal leaflets | WBL |
| Width of basal leaflet articulation | WBLa |
| Distance between basal leaflets | DbBL |
| Length of median leaflets | LML |
| Number of veins in median leaflet | NVML |
| Width of median leaflets | WML |
| Width of median leaflet articulation | WMLa |
| Distance between median leaflets | DbML |
| Length of apical leaflet | LAL |
| Width of apical leaflet | WAL |
| Width of apical leaflet articulation | WALa |
| Distance between of apical leaflets | DbAL |
| Length of pollen strobilus peduncle | LPSP |
| Diameter of pollen strobilus peduncle | DPSP |
| Length of pollen strobilus (fertile portion) | LPS |
| Diameter of pollen strobilus (fertile portion) | DPS |
| Length of microsporophylls | LMi |
| Width of microsporophylls | WMi |
| Horn length of microsporophylls | HLMi |
| Distance between horn of microsporophylls | DHMi |
| Length infertile portion of microsporophylls | LIpMi |
| Length of ovulate strobilus (fertile portion) | LOH |
| Diameter of ovulate strobilus (fertile portion) | DOS |
| Length of ovulate strobilus peduncle | LOSP |
| Diameter of ovulate strobilus peduncle | DOSP |
| Length of megasporophylls | LMe |
| Width of megasporophylls | WMe |
| Horn length of megasporophylls | HLMe |
| Distance between horn of megasporophylls | DHMe |
| Number of megasporophylls | NMe |
| Number of orthostichies | NOr |
| Number of megasporophylls per row | NMer |
| Length of seed | LS |
| Diameter of seed | DS |

Supplementary Data Table S4. Primers used in this study and GenBank Accession Numbers (Submission code 281408 and 2814697).

| Genome | Region | Forward primer | Reverse primer | Reference |
| --- | --- | --- | --- | --- |
| nrDNA | ITS, nuclear ribosomal ITS region | 5a CCTTATCATTTAGAGGAAGGAG3 | 4rev TCCTCCGCTTATTGATATGC3 | Pennisi, 2007; Nicolalde-Morejón et al., 2011 |
| ncDNA | GTP, GTP-binding protein Era mRNA | GTP_F2 TGATACWCCTGGTGTGAT | GTP_R2 CTCCATSTCCATATTTGGC | Salas-Leiva et al., 2013 |
| ncDNA | CyAG, MADS-box transcription factor family AGAMOUS | CeAG_F7 CCATTTCAGAGTCCAATTCTCAG | AGM3596_R CTTAGTGCGAAGAAACTGATTCTC | Salas-Leiva et al., 2013 |
| ncDNA | GroES, GroES-like zinc-binding alcohol dehydrogenase family protein | GroES_F1c CCAAGCTGATGATGGTAATTTC | GroES_R2 TACATGGTCWGCTCCTAA | Salas-Leiva et al., 2013 |
| ncDNA | HTS, Histidyl-tRNA synthetase | HTS_F1a AACTTCWGATGCTGTTGG | HTS_R3 CAGCACCATGACGCTTRAA | Salas-Leiva et al., 2013 |
| ncDNA | PEX4, peroxin4 | PEX4_F1 TCCAGCTAGCCATGACTGTTC | PEX4_R1 GGTTTTGACCCCTATTCGGTA | Salas-Leiva et al., 2014 |
| cpDNA | *matK*, maturase K | ATACCCCATTTTATTCATCC3 | GTACTTTTATGTTTACGAGC | Nicolalde-Morejón et al., 2011 |
| cpDNA | *psbK-psbI*, intergenic spacer | TTAGCCTTTGTTTGGCAA G | AGA GTTTGAGAGTAAGCAT | Pennisi, 2007; Nicolalde-Morejón et al., 2011 |
| cpDNA | trnS-trnG, intergenic spacer | GCCGCTTAGTCCACTCAGC | GAACGAATCACACTTTTACCAC | Sangin et al., 2008; Xiao et al., 2020 |

Supplementary Data Table S5. Molecular markers best-fit evolutionary model for trees inferred from Bayesian and Maximum Likelihood analyses.

| Analysis | Marker | Model |
| --- | --- | --- |
|  | ITS | HKY |
| Bayesian | GTP | 001202+F |
|  | CyAG | HKY |
|  | GroES | 010110+G+F |
|  | HTS | HKY |
|  | PEX4 | HYK |
|  | *matK* | 001202+F |
|  | trnS-trnG | TPM3uf+G |
|  | *psbK/psbI* | TPM1uf+G |
|  | ITS | HKY+F+R2 |
| Maximum Likelihood | GTP | TPM3+F+R2 |
|  | CyAG | HKY+F+R2 |
|  | GroES | TPM2+F+R2 |
|  | HTS | TPM3+F+R2 |
|  | PEX4 | HKY+F+R2 |
|  | *matK* | K3Pu+F+G4 |
|  | trnS-trnG | HKY+F+R2 |
|  | *psbK/psbI* | K3Pu+F+G4 |

Supplementary Data Table S6. Selected mean clade ages in millions of years ago and their 95% Highest Posterior Density (HPD) intervals.

| Calibration reference | Ages (Ma) | | |
| --- | --- | --- | --- |
|  | Median | Min | Max |
| *Zamia^a^* | 32.57 | 27.62 | 37.57 |
| *Bowenia^b^* | 42.02 | 35.91 | 48.31 |
| *Dioon^b^* | 53.33 | na | na |
| *Ceratozamia^c^* | 16.32 | 13.88 | 18.90 |
| Mexicana clade^c^ | 12.37 | 7.45 | 17.18 |
| Miqueliana clade^c^ | 12.02 | 7.62 | 16.15 |

^a^ Based on Calonje *et al*. (2019)

^b^ Based on Condamine *et al*. (2025)

^c^ Based on Medina-Villarreal *et al.* (2019)

na= not applicable

Supplementary Data Table S7. Bioclimatic variables and abbreviations according to WorldClim.

| Variable | Abbreviations |
| --- | --- |
| Annual Mean Temperature | BIO1 |
| Mean Diurnal Range (Mean of monthly (max temp - min temp) | BIO2 |
| Isothermality (BIO2/BIO7) (* 100) | BIO3 |
| Temperature Seasonality (standard deviation ×100) | BIO4 |
| Max Temperature of Warmest Month | BIO5 |
| Min Temperature of Coldest Month | BIO6 |
| Temperature Annual Range (BIO5-BIO6) | BIO7 |
| Mean Temperature of Wettest Quarter | BIO8 |
| Mean Temperature of Driest Quarter | BIO9 |
| Mean Temperature of Warmest Quarter | BIO10 |
| Mean Temperature of Coldest Quarter | BIO11 |
| Annual Precipitation | BIO12 |
| Precipitation of Wettest Month | BIO13 |
| Precipitation of Driest Month | BIO14 |
| Precipitation Seasonality (Coefficient of Variation) | BIO15 |
| Precipitation of Wettest Quarter | BIO16 |
| Precipitation of Driest Quarter | BIO17 |
| Precipitation of Warmest Quarter | BIO18 |
| Precipitation of Coldest Quarter | BIO19 |

Supplementary Data Table S9. Principal Component Analysis for all quantitative characters of the ovulate strobili in *Ceratozamia* species.

| Contributions | Component 1 | Component 2 |
| --- | --- | --- |
| LOH | 17.418578 | 0.870317602 |
| DOS | 14.271350 | 3.427217939 |
| LOSP | 4.877078 | 19.734359530 |
| DOSP | 8.290013 | 0.515415752 |
| LMe | 0.232052 | 0.009606243 |
| WMe | 1.062801 | 35.511570877 |
| HLMe | 1.569909 | 1.816244133 |
| DHMe | 1.594525 | 9.167472732 |
| NMe | 18.654921 | 2.757906534 |
| NOr | 14.874029 | 5.480350009 |
| NMer | 17.154744 | 0.709538650 |
| Eigen values | 4.55868830 | 1.43918821 |
| Percentage of variance | 41.4426209 | 64.19088 |

Supplementary Data Table S10. Principal Component Analysis for all quantitative characters of the pollen strobili in *Ceratozamia* species.

| Contributions | Component 1 | Component 2 |
| --- | --- | --- |
| LPSP | 18.436858 | 3.5634537 |
| DPSP | 1 5.922663 | 2.5384213 |
| LPS | 2.187704 | 7.5284938 |
| DPS | 12.966079 | 2.9203498 |
| LMi | 20.040348 | 0.2674891 |
| WMi | 12.838005 | 0.9511844 |
| DHMi | 3.242524 | 44.0260060 |
| HLMi | 4.090176 | 33.7127076 |
| LIpMi | 10.275642 | 4.4918945 |
| Eigen values | 3.8240619 | 1.4337070 |
| Percentage of variance | 42.48958 | 58.41965 |
